# Exploring Named Entity Recognition Potential and the Value of Tailored Natural Language Processing Pipelines for Radiology, Pathology, and Progress Notes in Clinical Decision Support: Quantitative Study

**DOI:** 10.2196/59251

**Published:** 2025-09-05

**Authors:** Veysel Kocaman, Fu-Yuan Cheng, Julio Bonis, Ganesh Raut, Prem Timsina, David Talby, Arash Kia

**Affiliations:** 1 John Snow Labs Inc Lewes, DE United States; 2 Institute for Healthcare Delivery Science Mount Sinai New York, NY United States; 3 Department of Anesteshiology, Perioperative and Pain Medicine Mount Sinai New York, NY United States

**Keywords:** natural language processing, electronic medical record, named entity recognition, clinical note, radiology report, pathology report, artificial intelligence, AI

## Abstract

**Background:**

Clinical notes house rich, yet unstructured, patient data, making analysis challenging due to medical jargon, abbreviations, and synonyms causing ambiguity. This complicates real-time extraction for decision support tools.

**Objective:**

This study aimed to examine the data curation, technology, and workflow of the named entity recognition (NER) pipeline, a component of a broader clinical decision support tool that identifies key entities using NER models and classifies these entities as present or absent in the patient through an NER assertion model.

**Methods:**

We gathered progress care, radiology, and pathology notes from 5000 patients, dividing them into 5 batches of 1000 patients each. Metrics such as notes and reports per patient, sentence count, token size, runtime, central processing unit, and memory use were measured per note type. We also evaluated the precision of the NER outputs and then the precision and recall of NER assertion models against manual annotations by a clinical expert.

**Results:**

Using Spark natural language processing clinical pretrained NER models on 138,250 clinical notes, we observed excellent NER precision, with a peak in procedures at 0.989 (95% CI 0.977-1.000) and an accuracy in the assertion model of 0.889 (95% CI 0.856-0.922). Our analysis highlighted long-tail distributions in notes per patient, note length, and entity density. Progress care notes had notably more entities per sentence than radiology and pathology notes, showing 4-fold and 16-fold differences, respectively.

**Conclusions:**

Further research should explore the analysis of clinical notes beyond the scope of our study, including discharge summaries and psychiatric evaluation notes. Recognizing the unique linguistic characteristics of different note types underscores the importance of developing specialized NER models or natural language processing pipeline setups tailored to each type. By doing so, we can enhance their performance across a more diverse range of clinical scenarios.

## Introduction

### Background

Clinical decision support (CDS) tools play a vital role in improving quality of care and patient safety by providing clinicians with real-time patient data and evidence-based guidance. In the 1960s and 1970s, paper-based diagnostic systems were introduced. They used some rules to match a patient’s symptoms with a set of possible diagnoses based on data from previous patients. One of the earliest examples of a paper-based diagnostic system was the Symptom Sorting Program, developed by Weed in 1968 [1]. In the 1980s, electronic medical records began to emerge, offering greater flexibility and more extensive datasets. Electronic medical records enabled clinical informaticists to collect and store patient data more efficiently, paving the way for the development of advanced CDS tools. One of the earliest examples of an electronic CDS tool was the HELP system, which was introduced in 1983 at the Latter-Day Saints Hospital in Salt Lake City, Utah [2]. These days CDS tools use a variety of technologies, including artificial intelligence, natural language processing (NLP), and machine learning, to analyze patient data and provide relevant recommendations to improve clinical effectiveness, optimize patient safety, and reduce the risk of errors [3].

There are several types of inputs coming from electronic health record (EHR) platforms that can be used to develop a CDS tool, including structured (eg, laboratory results and vital signs), semistructured (eg, nursing flow sheets), and unstructured (eg, clinical notes) data. Clinical notes and reports contain valuable patient data but can be challenging to analyze due to their unstructured nature. Clinical notes usually contain unorganized and inconsistent data with free-form text that make it difficult to extract the relevant information automatically. There are medical terms, abbreviations, and synonyms in clinical notes resulting in ambiguity and misinterpretation. Extracting clinical insights from the large volume of unstructured data can be time consuming, which can have a significant impact on the real-time applicability of decision support tools [4].

NLP is a branch of artificial intelligence focused on enabling computers to understand, interpret, and generate human language. In the context of clinical notes, NLP techniques can be used to extract information from unstructured text, including named entity recognition (NER), which identifies and extracts entities such as diseases, signs and symptoms, and treatments [5]. In recent years, deep learning–based NER models in the clinical domain have reached performance levels that make them suitable for developing CDS tools with sufficient reliability [6]. For instance, Spark NLP [7] is an open-source library built on Apache Spark that provides various tools and pretrained models for NLP tasks, such as NER [8,9], entity assertion, and entity relationship extraction. However, developing and deploying such CDS tools pose various challenges that require careful consideration [10]. In this paper, we discuss some of the key challenges and best practices for developing and deploying NER-based CDS tools in clinical settings.

### Challenges of NER-Based CDS Tools

There are 5 main areas of challenges in the development and deployment of NLP-based CDS tools: note ingestion and curation, technology, computational workflow, clinical workflow, and user adoption. In this paper, our main focus is on data curation, technology, and computational workflow. Clinical note ingestion and curation can affect the performance of NLP models, and the choice of technology can impact scalability and reproducibility. To address the challenges we are sharing the best practices coming from the lessons we learned from our practical experiences.

## Methods

### Ethical Considerations

Data used during this study was managed following Health Insurance Portability and Accountability Act–compliant procedures based on retrospective anonymized electronic healthcare records datasets. Given the analysis was purely focused on computational performance and overall performance of information extraction models and not related with any clinical consideration we concluded that this study did not involve human subjects.

### CDS Application Architecture

**Figure 1:**
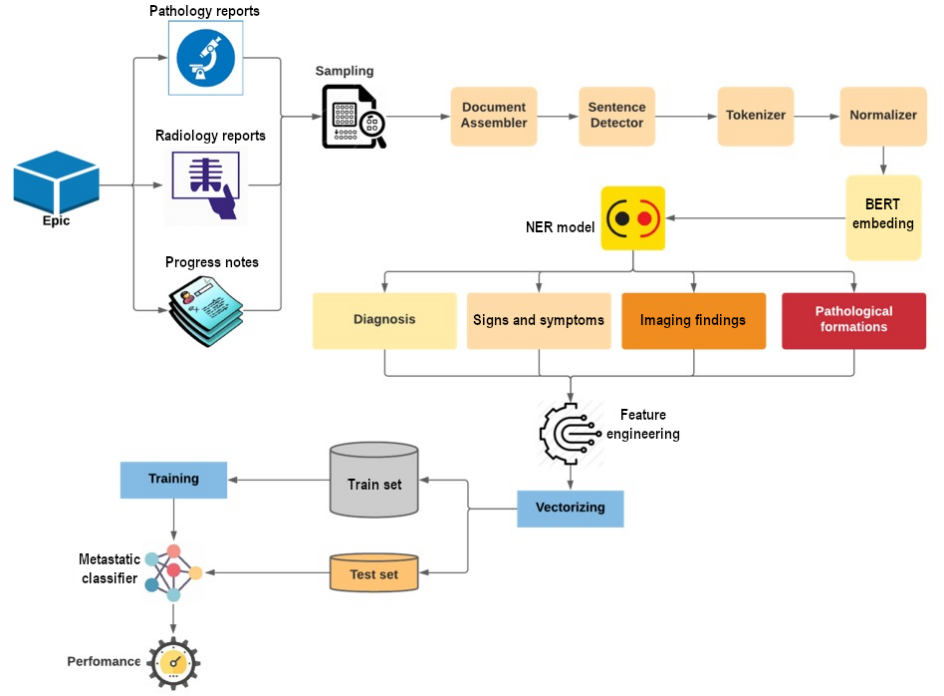
Computational flow.

The work presented here is part of a larger CDS system designed to enhance patient care through timely data curation and patient-level risk stratification, with its computational flow shown in Figure 1. A key component of this system was the NER pipeline, which focuses on extracting essential clinical entities from unstructured notes, including progress notes, radiology reports, and pathology reports. Once relevant entities were identified through NER, these extracted entities were subsequently used as inputs to machine learning classifiers that support clinical decision-making, offering actionable insights to clinicians at the point of care.

The NER pipeline itself comprises 2 main components: the *note ingestion pipeline* and the *NLP pipeline*. This section provides an overview of the architecture of this NER pipeline, which was implemented for oncology and psychiatry CDS tools to generate risk scores for metastatic disease and the likelihood of developing episodes of aggression. However, the scope of this paper is limited to presenting the entity extraction process without going into the specifics of the downstream classification components that use this information. The description and validation of these downstream components that complete the CDS tool will be the subject of future publications.

### Note Ingestion Pipeline

Different clinical notes (eg, progress note, care note, and consult note) and reports (eg, radiology impression and pathology report) were ingested from various EHR modules (Epic) such as Clinical Notes, Radiology Information System (RIS), and pathology information system. A cross-platform interface engine (Mirth Connect) [11] was used for note interfacing. Mirth stored the Health Level 7 (HL7) messages in a NoSQL database (MongoDB), and a Health Level 7 parser (Smolder) [12] extracted and decrypted these notes before saving them into the NoSQL database.

A Spark service then read the raw notes and stored them in a Spark Dataframe, creating a unified view at the desired level, which can be patient, visit, or bed level, based on the specifications of the use cases. Similar to most data elements in an EHR, such as laboratory results and vital signs, clinical notes and reports are considered observational data. To use them as inputs for modeling, specific sampling logic needs to be applied to optimize the learning rate during the training phase.

The following sampling logic was applied to select relevant clinical notes and compile a comprehensive text corpus for the NLP pipeline. Notes were first organized by type, and then, a random sampling was performed within each category to ensure a diverse selection. Progress care notes, which are typically longer and contain more clinical entities, were oversampled to capture a wide variety of detailed clinical information. Conversely, radiology and pathology reports, known for their brevity, were undersampled to maintain their typical representation in clinical workflows. This stratified approach created a balanced training dataset that exposed the model to a representative mix of clinical scenarios while preserving the natural variation in note types.

### NLP Pipeline

The NLP pipeline is a series of interconnected modules designed and developed to process clinical notes and extract predefined sets of medical terms using pretrained NER models. The pipeline consists of several key components, including the document module, sentence module, tokenizer module, embedding model, and NER models.

The document module handles the input of clinical notes and ingests them into a Spark DataFrame. The sentence module then further segments these documents into separate sentences, enabling more granular analysis. The tokenizer module plays a crucial role in splitting sentences into individual words or tokens, facilitating subsequent analysis and processing. The embedding module focuses on transforming the tokenized words into numerical representations, often leveraging techniques such as word embeddings or contextualized embeddings. This step allows the pipeline to capture semantic meaning and context within the text. Finally, the NER module is a vital component that identifies and classifies named entities within clinical notes, such as diagnoses, signs and symptoms, medications, and electrocardiography findings. This module uses pretrained NER models to accurately recognize and extract relevant entities from the text.

To execute the NLP pipeline efficiently, it is often implemented using Spark NLP [13], a powerful NLP library built on top of Apache Spark [14]. This choice allows the pipeline to leverage the distributed computing capabilities of Apache Spark, enabling faster and scalable processing of large volumes of clinical notes.

By using this NLP pipeline with its various modules and pretrained models [15], health care professionals and researchers can automate the extraction of important information from clinical notes, such as medical conditions, treatments, patient outcomes, and even some complex information (eg, response to treatment). This approach streamlines the analysis process, facilitates research, and enhances decision-making in the health care domain.

### Performance Measurement

In this section, we present the methodology and metrics used to measure the performance of the note ingestion and curation pipeline and NLP pipeline. The server on which we ran the pipelines was an Intel Xeon (R) Gold 5120 central processing unit (CPU) @ 2.20 GHz with 56 processors, 14 CPU cores, and 187 Gb memory size. We ingested and curated progress and care notes, radiology reports, and pathology reports. We split them into 5 batches, each with notes of 1000 unique patients, and measured the number of notes and reports per patient, the number of sentences, token size, runtime, CPU use, and memory use per note type.

We also assessed the precision (positive predictive value) of the pretrained NER models in identifying 4 entities: disease, symptom, drug, and procedure. To achieve this, a clinical expert reviewed a random sample of clinical notes containing entities identified by the pretrained NER models, classifying each prediction as a true positive or false positive. The assertion model developed specifically for this project was evaluated by a clinical expert, who reviewed 342 randomly selected sentences in which an entity was identified and marked as present or absent. The expert then confirmed whether the assertion generated by the model was correct.

## Results

### Clinical Note Characteristics

Our analysis of a corpus comprising 138,250 notes from 5000 patients randomly selected from those aged >18 years admitted to the Mount Sinai Hospital revealed distinct characteristics for each type of note.

The distribution of notes per patient was positively skewed across all note types, with pathology notes ranging from 1 to 17, progress care notes from 1 to 99, and radiology notes from 1 to 88. The median number of notes per patient was 1 (interdecile range 1-2) for pathology notes, 15 (interdecile range 5-47) for progress care notes, and 3 (interdecile range 1-11) for radiology notes. Moreover, 90% (4500/5000) of the patients had 1 or 2 pathology notes, 5 to 47 progress care notes, and 1 to 11 radiology notes. As shown in Table 1, there were differences in the average number of notes per patient among the different types of notes. Progress care notes were the most prevalent, averaging 21.16 (95% CI 20.63-21.69) notes per patient, followed by radiology notes with 5.02 (95% CI 4.85-5.19) and pathology notes with 1.47 (95% CI 1.44-1.50).

Upon closer examination of the notes themselves, we found that pathology notes were the highest in terms of the number of sentences, averaging 23.85 (SD 22.78; median 17) sentences per note, followed by progress care notes with 17.14 (SD 21.57; median 11) sentences per note. In contrast, the number of individual sentences in radiology notes was much lower, with an average of 4.77 (SD 6.87; median 3) sentences per note.

However, our analysis of sentence length based on note type revealed that progress and care notes contained longer sentences, averaging 36.46 (SD 75.57; 95% CI 36.35-36.57) tokens per sentence. In comparison, radiology and pathology notes were less than half the length, with 11.83 (SD 8.56; 95% CI 11.79-11.87) and 12.16 (SD 8.83; 95% CI 12.11-12.21) tokens per sentence, respectively. Although the difference in sentence length between pathology and radiology notes was statistically significant, the practical implications were minimal.

Interestingly, when examining the median number of tokens per sentence, the differences between pathology (10 tokens), progress care (12 tokens), and radiology notes (10 tokens) were not significant in practical terms. The difference in means was driven by the existence of some long sentences in the progress care notes, with an interdecile range of 4 to 85 tokens. For pathology and radiology notes, the ranges were 3 to 23 and 3 to 22 tokens, respectively.

Statistically significant differences were observed in the number of clinical entities per sentence and the proportion of tokens constituting a clinical entity (clinical entity density).

Progress and care notes exhibited the highest clinical entity density, with 8.01 (SD 24.05; 95% CI 7.98-8.05) clinical entities per sentence and 21.97% (SD 1.66%; 95% CI 21.97-21.99%) of tokens forming a clinical entity chunk. Radiology notes contained a quarter of the number of clinical entities per sentence (2.06; SD 2.65; 95% CI 2.05-2.08) but had a comparably lower entity density (16.92%; SD 6.47%; 95% CI 16.88-17.04%). Conversely, pathology notes had the lowest number of clinical entities per sentence (0.53%; SD 1.07; 95% CI 0.53-0.54) and the smallest proportion of tokens as part of a clinical entity chunk (4.52%; SD 1.31%; 95% CI 4.49-4.55%).

Again, the higher average number of entities found in the progress and care notes, when compared to radiology notes, was driven by a subgroup of sentences with an unusually high number of clinical entities. The median number of clinical entities per sentence was the same for both types of notes (2 entities). However, the range was wider for progress and care notes (0-15 clinical entities per sentence) compared to radiology notes (0-5 clinical entities per sentence).

**Table 1:** Descriptive analysis of the corpus compiled from 5000 patients. The interdecile range is represented by the 10th and 90th percentiles.

	Pathology	Progress care	Radiology
Patients, n	5000	5000	5000
Notes, n	7374	105,799	25,098
Sentences, n	175,874	1,813,194	119,796
Tokens, n	2,080,590	66,109,058	1,456,722
Entities, n	94,075	14,531,843	247,319
Notes per patient, mean; SD (95% CI)	1.47; 1.08 (1.44-1.50)	21.16; 19.12 (20.63-21.69)	5.02; 6.13 (4.85-5.19)
Sentences per note, mean; SD (95% CI)	23.85; 22.78 (23.33-24.37)	17.14; 21.57 (17.01-17.27)	4.77; 6.87 (4.69-4.86)
Tokens per sentence, mean; SD (95% CI)	11.83; 8.56 (11.79-11.87)	36.46; 75.57 (36.35-36.57)	12.16; 8.83 (12.11-12.21)
Entities per sentence, mean; SD (95% CI)	0.53; 1.07 (0.53-0.54)	8.01; 24.05 (7.98-8.05)	2.06; 2.65 (2.05-2.08)
Notes per patient, mean (interdecile range)	1 (1-2)	15 (5-47)	1 (1-11)
Sentences per note, mean (interdecile range)	17 (8-46)	11 (1-42)	3 (1-9)
Tokens per sentence, mean (interdecile range)	10 (3-23)	12 (4-85)	10 (3-22)
Entities per sentence, mean (interdecile range)	0 (0-2)	2 (0-15)	2 (0-5)
Clinical entity density %; SD (95% CI)	4.52; 1.31 (4.49-4.55)	21.98; 1.66 (21.97-21.99)	16.92; 6.47 (16.88-17.04)

### NER Performance

We used a set of pretrained NER models to identify 4 types of clinical entities: diseases (n=340), drugs (n=173), procedures (n=281), and symptoms (n=4412). As we only reviewed the actual output of the NER models and did not annotate a gold standard corpus, other metrics are not available for evaluation. The highest precision was achieved for procedure entities (0.989; SD 0.098; 95% CI 0.977-1.000), followed by diseases (0.918; SD 0.277; 95% CI 0.888-0.947) and symptoms (0.929; SD 0.254; 95% CI 0.922-0.937). Drug entities exhibited significantly lower precision (0.821; SD 0.382; 95% CI 0.764-0.878). We also implemented an assertion model to categorize whether an entity was present or absent (n=342). The model demonstrated a recall (sensitivity) of 0.922 (SD 0.264; 95% CI 0.894-0.950), a precision (positive predictive value) of 0.828 (SD 0.377; 95% CI 0.788-0.868), a specificity of 0.866 (SD 0.340; 95% CI 0.830-0.902), a negative predictive value of 0.941 (SD 0.241; 95% CI 0.915-0.966), an accuracy of 0.889 (SD 0.311; 95% CI 0.856-0.922) and an *F*_1_-score of 0.872. The system used in this experiment was equipped with an Intel Xeon Gold 5120 CPU, operating at a speed of 2.20 GHz. It featured 56 processors, arranged into 14 CPU cores, each running at approximately 2599.877 MHz. The setup also included a substantial memory capacity of 187 GB, ensuring sufficient resources for handling the data processing demands. The compute time measurements presented are specific to the previously described hardware setup and may vary across different computational environments.

We opted for a CPU-based setup instead of a graphics processing unit due to the highly sensitive nature of the data derived from EHRs. In many organizations, it is preferable to run such processes on premises to ensure data security and confidentiality, minimizing the need to share data with external infrastructures. This practical constraint often means that graphics processing units are not easily available, making a CPU setup both a feasible and secure option for processing sensitive clinical information directly within the organization’s own hardware environment.

The process involved dividing each note type into 5 mini-batches, with each batch containing all notes from 1000 patients. We further partition each mini-batch into 100 partitions. This results in each partition containing 10 sequences. This methodology significantly enhances our capability for parallel processing. The logging frequency was set to every 5 seconds. In terms of runtime per mini-batch, radiology and pathology notes averaged 24 and 33 seconds, respectively. Contrastingly, the average runtime for progress and care notes was significantly higher, clocking in at 1498 seconds. This means that progress and care notes required approximately 42 times more time than pathology notes and approximately 56 times more than radiology notes.

One key finding was that the average number of tokens in progress and care notes was approximately 30 times more than in pathology notes and approximately 31 times more than in radiology notes. Hence, this substantial increase in runtime for progress and care notes was anticipated. The experiment clearly demonstrates a linear relationship between runtime and the size of the notes. The mean, SD, and percentile values of computing time for each type of clinical note are presented in Table 2.

**Figure 2:**
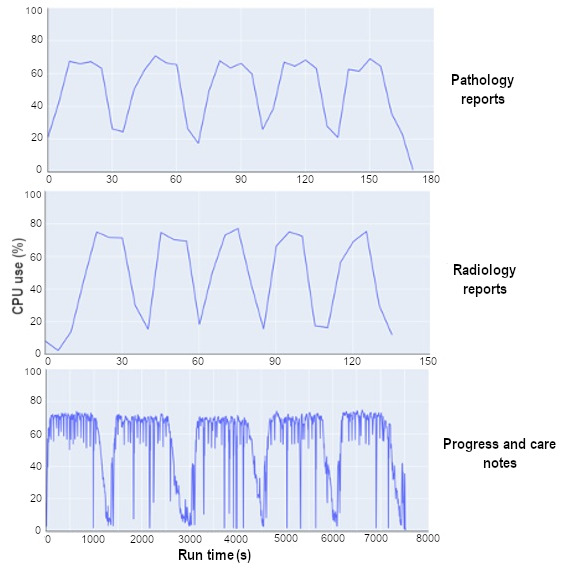
Central processing unit (CPU) use over runtime by note type.

Figure 2 presents a graph of CPU use. We noticed similar patterns of CPU use for both radiology and pathology notes, fluctuating between 20% and 80%. By contrast, CPU use for progress and care notes ranged from 5% to 80% but consistently remained at the higher end of this spectrum. This was an anticipated outcome, given that the token size of progress and care notes is considerably larger than that of the other note types.

**Table 2:** Computational times.

Note type	Mean (SD)^a^	Minimum	Percentile					Maximum
			10th	25th	50th	75th	90th	
Pathology	35.4 (7.3)	30	30	31	33	35	43	48
Progress care	1498.0 (132.0)	1354	1374	1405	1505	1530	1630	1696
Radiology	26.6 (6.4)	23	23	23	24	25	33	38

^a^Seconds required to process all the clinical notes of each type for 5000 patients (5 batches of 1000 patients).

## Discussion

### Principal Findings

The development and deployment of CDS tools using the NER approach presents numerous challenges and opportunities. We believe that our work contributes to the knowledge in this field in several significant ways.

In contrast to previous studies focused on specific clinical note types, our analysis includes progress care notes, radiology reports, and pathology notes, enabling a comprehensive comparison of linguistic features, entity density, and NER performance across these distinct categories. The findings emphasize the necessity of tailored NLP pipelines, as progress care notes, for instance, require significantly greater computational resources and processing time than radiology or pathology notes. The notably higher clinical entity density observed in progress care notes, compared to radiology and pathology reports, further highlights the variability in NER performance across clinical domains and reinforces the need for context-specific optimization approaches. This underscores a gap in existing NER optimization strategies that general-purpose models may fail to address.

Moreover, our study extends beyond traditional accuracy metrics by providing detailed computational insights, including compute times, CPU use, and memory use, which are essential for optimizing real-time CDS applications.

### Characteristics of Different Clinical Notes

This study provides a comprehensive understanding of the characteristics and linguistic composition of different types of clinical notes. Our analysis revealed long-tail distributions in terms of notes per patient, note length, and clinical entity density. Therefore, the 90th percentile could serve as a practical benchmark when determining the necessary context length for various NLP tasks on this type of clinical note–based corpus. It is more practical and logical to design NLP systems to accommodate 90% of the cases, excluding outliers with an unusually large number of notes per patient or exceptionally lengthy notes and sentences.

We identified significant differences in note length, sentence length, and the presence and density of clinical entities among the note types. These differences likely stem from the distinct clinical contexts in which these notes are generated. One possible explanation is the difference in focus between patient care specialties and diagnostic specialties. For example, progress care notes are typically authored by clinicians involved in ongoing patient care, such as internal medicine or critical care physicians. These notes are more narrative and detailed, documenting the patient’s daily progress, treatment plans, and responses to therapy. This structure results in a greater number of clinical entities associated with patient management, while the longer sentence and note lengths reflect the complexities of continuous care.

In contrast, radiology and pathology reports are usually created to interpret specific tests or procedures. Therefore, they are shorter and more structured, with fewer entities per sentence, as they summarize diagnostic findings rather than provide a holistic account of the patient’s clinical status. The lower density of clinical entities in these notes likely reflects the narrower focus on reporting test results instead of capturing comprehensive patient management. It is also plausible that for other entity types more pertinent to pathology reports, such as anatomical structures, sizes, or locations, the entity density would be higher within this specific type of notes.

Patients typically had a single pathology note, and only 10% (4500/5000) of patients had >2 pathology reports, likely reflecting the definitive nature of pathology results in diagnosing a medical condition. In contrast, the median number of radiology reports was 3. This observation suggests that medical imaging tests are used not only for diagnostic purposes but also for reassessment and follow-up.

When analyzing the distribution of sentence lengths, we observed peaks at specific values that did not align with the expected statistical distribution. A qualitative analysis of these sentences revealed that they were primarily related to copied or templated content. For instance, within the corpus, 7521 sentences consisted of the following 20 tokens: “Attending physician note: \n I have personally reviewed the images and resident’s interpretation \n thereof and agree with the findings.” This finding is consistent with the well-documented phenomenon of redundancy in clinical notes [16,17].

### Optimization Strategies and Workload Distribution

#### Overview

In NLP, particularly in deep learning–based NER systems such as bidirectional long short-term memory and conditional random fields, sentence length plays a pivotal role. This is because sentences are the primary units of processing in such architectures, contrasting with large language models where the context window can encompass longer text spans. Consequently, for deep learning NER, overly long or short sentences can impact the model’s ability to accurately recognize and categorize entities, emphasizing the need for optimal sentence segmentation.

One significant finding of this study is the variation in processing times across different types of clinical reports, as described in Table 2. This variation indicates the complexity and diverse nature of different report types and highlights the need for tailored optimization strategies to improve efficiency. However, progress and care notes exhibited considerably higher runtime. This discrepancy suggests that progress and care notes may contain more extensive and detailed information compared to other report types, which requires greater memory allocation for processing. The observed CPU use across all 3 processing tasks, including pathology, radiology, and progress and care notes, remained relatively consistent, as depicted in Figure 2. This consistent CPU use indicates a balanced workload distribution during the processing of clinical reports. However, further optimization could be explored to ensure efficient use of computational resources and enhance overall system performance.

These findings emphasize the need for tailored optimization strategies for different report types to achieve optimal processing times and resource use. In addition, it underscores the importance of allocating sufficient memory resources, particularly for more complex report types such as progress and care notes. There are some suggestions related to optimization strategies, inlucding the following:

#### NoteType-Specific Preprocessing

The goal of this approach is to identify and prioritize key sections or entities within each report type to streamline the processing pipeline. Different note types varied significantly in their entity densities and sequence lengths. This variety not only impacts processing times but also requires tailored preprocessing strategies.

Pathology and radiology notes, while generally shorter, may not necessarily contain fewer entities; rather, they tend to have a higher density of specific entities such as anatomical sites, size, and histological types, as covered in this paper. Conversely, longer progress care notes, replete with intricate details of patient care, pose a greater challenge due to their extended runtime and larger data volumes. Therefore, computational optimization strategies for these notes must account not just for the type of document but also for the specific entities targeted for extraction. Given this context, these progress notes necessitated nuanced preprocessing, including segmentation into smaller parts, to ensure efficient handling.

Therefore, the key takeaway is the need for flexible preprocessing strategies, adaptable to the specific characteristics of each note type, to ensure both efficiency and accuracy in processing diverse clinical notes.

#### Algorithmic Optimization

The goal of this approach is to explore and fine-tune NER models to suit the specific requirements of each note type. Different report types may have distinct patterns or structures, and optimizing the algorithms accordingly can lead to more accurate and efficient processing. This may involve leveraging domain-specific knowledge, incorporating machine learning techniques, or adopting advanced NER models to enhance performance.

#### Resource Allocation Optimization

It is advisable to allocate computational resources, such as memory and processing power, based on the requirements of each report type. Because different report types may have varying complexities and information densities, it is crucial to optimize resource allocation accordingly. If the progress care notes contain a greater number of tokens per sentence (ie, longer sequences), this leads to an extended runtime. In such a situation, increasing computational resources and memory allocation could prove to be an effective strategy. Alternatively, we could also consider decreasing the mini-batch size and carrying out distributed processing for each. This method could potentially mitigate the runtime bottleneck issue. Such strategies become particularly vital when these tools are implemented in real-time or near–real-time environments.

### Performance of the NER Models

The implemented NER models demonstrated promising precision in identifying clinical entities, with procedures showing the highest precision. The assertion model also exhibited a high level of accuracy in categorizing the presence or absence of entities, highlighting the potential of NLP tools in processing and comprehending complex clinical data.

Recall (sensitivity) was not evaluated in this project for NER models, as it would have required a significant manual annotation effort beyond the primary objectives of the study. Pretrained NER models used in this study have been reported to achieve high *F*_1_-scores (0.96 for disease and drug entities and 0.86 for procedure and symptom entities [8]), indicating presumably adequate recall for our research aims. Regarding the potential impact of recall on comparing entity density across different document types (eg, pathology, progress notes, and radiology), we hypothesize that recall performance is likely consistent across these document types. This consistency is expected, as the NER model was pretrained on data from a wide variety of clinical documents [9], suggesting that any recall-related misclassifications would be nondifferential across document types. Therefore, we do not anticipate that variations in recall would significantly affect our conclusions on entity density differences among document types.

### Conclusions

Given the promising findings of our study, it is crucial to pursue further research in this domain. We encourage additional exploration of the application and optimization of NLP tools in clinical contexts. Future studies should consider expanding the range of clinical entities under consideration and further investigate the phenomena of redundancy and the effects of templated content within clinical notes.

Moreover, further research could analyze different types of clinical notes that were not included in our study, such as discharge summaries or psychiatric evaluation notes. Understanding the linguistic characteristics of these notes could expand the applicability of NLP tools and enhance their performance across a broader range of clinical scenarios.

In conclusion, this study provides valuable insights into the development and deployment of CDS tools using NER. The variation in processing times, memory use, and CPU use across different report types underscores the importance of tailored optimization strategies and resource allocation. These lessons contribute to the overall knowledge base for designing effective CDS tools and inform future research and development efforts in this domain.

## Abbreviations

CDS: clinical decision support
CPU: central processing unit
EHR: electronic health record
NER: named entity recognition
NLP: natural language processing
